# Existing and Evolving Landscape of Medulloblastoma: Towards Optimization and Personalization

**DOI:** 10.3390/diagnostics14060598

**Published:** 2024-03-12

**Authors:** Tejpal Gupta, Abhishek Chatterjee

**Affiliations:** Department of Radiation Oncology, ACTREC/TMH, Tata Memorial Centre, HBNI, Kharghar, Navi Mumbai 410210, India

Advances in diagnostic imaging, pathology, and molecular biology coupled with improvements in neurosurgical approaches, radiotherapeutic techniques, and systemic therapies over the last two decades have vastly improved survival outcomes for medulloblastoma, the most common childhood malignant tumor [1] involving the brain and central nervous system (CNS). 

One hundred years ago, Harvey Cushing and Percival Bailey presented their research on the tumor ‘spongioblastoma cerebelli’ at an American Neurological Association meeting [2], describing a ‘small round blue-cell tumor’, which they believed arose from embryonal rests of undifferentiated cells within the roof and ependymal lining of the fourth ventricle. The term spongioblastoma aptly described the soft and suckable consistency of the tumor, but was later replaced by ‘medulloblastoma’ based on the premise that medulloblasts are one of five types of stem cells in the primitive neural tube. In the 1950s, the high propensity of the disease to spread along the neuraxis via the cerebrospinal fluid (CSF) pathways [3] and the realization of the susceptibility of these primitive embryonal cells to X-rays [4] ushered in an era of adjuvant craniospinal irradiation (CSI) for sustained tumor control [5]. The last forty years have witnessed the evolution of systemic chemotherapy approaches in the curative-intent contemporary management of medulloblastoma [6]. From initially being considered a nearly fatal disease without any adjuvant therapy despite gross total resection to its current status as a childhood cancer that is highly curable with post-operative risk-stratified adjuvant radio(chemo)therapy (Figure 1), the treatment of medulloblastoma has emerged as one of the most remarkable success stories in pediatric neuro-oncology [7]. Aggressive multi-modal management cures a substantial proportion of patients with medulloblastoma; however, such therapies are associated with a significant burden of dose-dependent treatment-related toxicities and resultant chronic health conditions that have a detrimental impact on health-related quality of life, including long-term survivorship [8,9].

Ground-breaking global research conducted by various dedicated neuro-oncology groups working in parallel has finally led to the consensus that medulloblastoma is a heterogenous disease [10] comprising four broad molecular subgroups—wingless (WNT), sonic hedgehog (SHH), Group 3, and Group 4 medulloblastoma, respectively—with subgroup-specific developmental origins, distinct clinico-demographic presentation, unique genetic profiles, and diverse clinical outcomes; this has led to the incorporation of molecular/genetic information in contemporary risk-stratification schema [11], as well as the fifth edition of the World Health Organization (WHO) classification of tumors involving the brain and CNS (WHO CNS 5) [12]. The existence of heterogeneity within each broad subgroup has resulted in a consensus on second-generation molecular subgrouping [13], with various subtypes being described within each broad subgroup further increasing the complexity of diagnosis and classification.

This Special Issue on medulloblastoma provides a broad overview of diagnostic imaging, including the evolving role of radiomics/radiogenomics; discusses its basic pathobiology and molecular genetics, including contemporary risk stratification; summarizes the existing evidence base for current standard-of-care therapies, including the emerging role of proton beam therapy; and navigates through the clinical trial landscape based on molecular subgrouping for the optimization of adjuvant therapy. The translation of emerging biological and molecular information into tangible benefits for patients in the clinic represents a formidable challenge for the pediatric neuro-oncology community. Rechberger and colleagues (contributor 1) discuss the four broad molecular subgroups of medulloblastoma, provide recent updates on its molecular landscape and complexity, explore the role of epigenetic regulation and the mechanism of resistance to therapy, and delve into the latest developments in targeted therapy and immunotherapy. The recommended gold-standard method for molecular subgroup assignment, i.e., DNA methylation profiling [14], has issues in terms of its availability, affordability, and accessibility. The differential expression of a set of protein-coding genes using real-time reverse transcriptase polymerase chain (RT-PCR) for molecular subgroup assignment in medulloblastoma [15,16], though robust, can be a difficult test to perform, particularly in resource-constrained low–middle-income countries (LMICs) [17]. Therein lies the utility of extracting semantic [18] and/or radiomic features [19] from pre-operative magnetic resonance imaging (MRI), which can potentially predict the broad molecular subgroups with reasonable accuracy [18,19,20]. Further confirmation of molecular subgrouping can be achieved using simpler, cheaper, and more widely available tool like immunohistochemical panels [17,21] of commercially available antibodies. Ongoing research suggests that such radiomic features can also be used either alone or in combination with clinical features to predict survival in medulloblastoma. Ismail et al. (contributor 2) provide a comprehensive overview of radiomic and radiogenomic analysis for tumor segmentation, molecular subgroup classification, and survival prognostication in the context of pediatric medulloblastoma. Further, they shed light on existing challenges in current radiomic approaches and highlight future directions and opportunities for more refined and nuanced computational analysis. Radiotherapy (RT) in the form of CSI plus boosts to primary-site and metastatic deposits (if any), delivered using high-energy photons from a linear accelerator, forms the backbone and cornerstone of adjuvant therapy in non-infantile medulloblastoma. The planning and delivery of CSI remains one of the most difficult and challenging processes in RT due to the target volume being large, irregularly shaped, complex, and surrounded by normal critical structures that need to be spared the detrimental impact of irradiation. The advent of intensity-modulated radiation therapy (IMRT), particularly rotational IMRT techniques, due to advances in physics and technology have vastly improved the process of the photon-based treatment planning, delivery, and verification of CSI in contemporary neuro-oncologic practice [22,23]. The emergence of proton-beam therapy with superior depth–dose characteristics (compared to photons) and resultant improvements in dosimetry provides an opportunity to further sculpt the dose away from surrounding normal structures conforming to the defined target volumes, thereby further reducing the incidence and severity of treatment-related toxicity in clinical practice [24]. Das and associates (contributor 3) report a preliminary study from South East Asia’s first proton therapy centre on the topic of image-guided, intensity-modulated proton therapy (IMPT) using pencil-beam scanning technology in their patient cohort of medulloblastoma (n = 40), with a focus on dosimetry, acute toxicity, and early survival outcomes. While the dosimetric superiority of protons compared to photons is now well established, an unequivocal clinical benefit is yet to be demonstrated in the context of a randomized controlled trial. Furthermore, the availability, accessibility, and affordability of modern proton beam therapy technology remain major challenges throughout the world, especially in LMIC settings. 

With the development of systemic chemotherapy protocols aimed at improving survival and reducing the long-term toxicity of high-dose CSI, the management of medulloblastoma in children has dramatically changed over the past four decades [6]. This includes the use of irradiation-sparing approaches, such as intraventricular methotrexate-based chemotherapy in medulloblastoma with extensive nodularity (MBEN) and high-dose chemotherapy with autologous stem-cell rescue in infantile (<3–5 years) medulloblastoma. The use of targeted therapy such as SHH inhibitors also represents as attractive paradigm in patient with SHH-subgroup medulloblastoma. The evolution of systemic therapies, including chemotherapy, targeted therapy, and immunotherapy, is reviewed in detail by Mushtaq and colleagues (contributor 4). Of all the subgroups, patients with WNT-pathway medulloblastoma have the best survival outcomes (5-year survival exceeding 90%), with appropriate radio(chemo)therapy leading to systematic attempts at treatment de-intensification to reduce the late morbidity associated with this treatment [25]. Prados (contributor 5) reviews the clinical trial landscape in medulloblastoma through a brief summary of recently reported randomized controlled trials in an upfront setting and introduces some of the ongoing prospective studies integrating molecular/genetic information with standard clinico-radiological factors for risk stratification. The incurable nature of relapsed medulloblastoma with current salvage regimens and exciting novel combinatorial approaches targeting multiple pathways, including cytotoxic therapies, small-molecule inhibitors, and immunotherapy strategies to improve outcomes in recurrent/progressive disease, are also briefly discussed. Finally, Mani et al. (contributor 6) report a single-institution audit of clinical outcomes in a molecularly characterized cohort of WNT-subgroup medulloblastoma (n = 67), confirming excellent long-term survival with adequate and appropriate upfront treatment using post-operative adjuvant radio(chemo)therapy. This retrospective audit also briefly discusses the premature termination of two de-escalation studies in low-risk WNT-activated medulloblastoma (one omitting upfront CSI and another using a post-surgery primary chemotherapy-only approach) due to an unacceptably high risk of relapse. 

In conclusion, our fundamental understanding of medulloblastoma biology has vastly improved over time, leading to continued refinements in risk stratification and prognostication. In parallel, aggressive multi-modal therapy cures a substantial proportion of children with medulloblastoma; however, these long-term survivors experience various late effects of treatment affecting their quality of life. The next decade of research in medulloblastoma should focus on finding the right balance between survival and survivorship through the optimization and personalization of therapy.

## Figures and Tables

**Figure 1:**
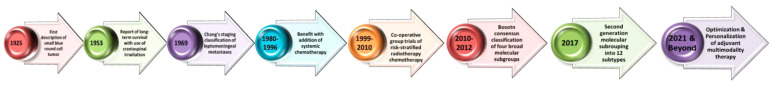
Milestones and timeline of the evolving landscape of medulloblastoma.
